# Effect of adding dexmedetomidine on two methods of labor analgesia via epidural among parturient patients

**DOI:** 10.22088/cjim.14.4.648

**Published:** 2023

**Authors:** Amineh Shafeinia, Faranak Rokhtabnak, Salume Sehat Kashani, Golnosh Khosravian, Poupak Rahimzadeh

**Affiliations:** 1 Department of Anesthesiology , Shahid Akbarabadi Hospital, School of Medicine, Iran University of Medical Sciences, Tehran, Iran; 2 Department of Anesthesiology, Firoozgar Hospital, School of Medicine, Iran University of Medical Sciences, Tehran, Iran; 3 Department of anesthesiology, Rassol Akram Hospital, School of Medicine, Iran University of Medical Sciences, Tehran, Iran; 4 Pain Research Center, Department of Anesthesiology, School of Medicine, Iran University of Medical Sciences, Tehran, Iran

**Keywords:** PIEB, CEI, Labor analgesia, Epidural dexmedetomidine.

## Abstract

**Background:**

Continuous epidural infusion (CEI) has been an optimal and acceptable technique for inducing epidural anesthesia. This study compared two methods of programmed intermittent epidural bolus (PIEB) with CEI in labor analgesia among patients receiving epidural dexmedetomidine.

**Methods:**

This study was a randomized clinical trial. The target population was term women candidates for epidural anesthesia. After selection of sample size based on inclusion criteria, a total of 3 cc of dexmedetomidine (0.5 µg/ml) and Ropivacaine 0.1% was injected. Furthermore, 5 ml was injected as a loading dose of dexmedetomidine 0.5 µg /ml and Ropivacaine 0.1%. Then the pain score was recorded. SPSS software Version 23 was used for statistical analysis of data.

**Results:**

The neonatal Apgar score in PIEB method was more improved (P = 0.003) and the use of assisted delivery tools such as vacuum, in PIEB method was reduced.

(p=0.038) Duration of the first phase of the labor in this method was more reduced than CEI.(p=0.015) Patients in the group undergoing epidural anesthesia by PIEB method were associated with a higher level of satisfaction with the delivery process (p < 0.05) than patients undergoing CEI protocol.

**Conclusion:**

PIEB method is associated with further improvement in neonatal Apgar score and maternal outcomes (reduction in the duration of the first phase of labor and no need to use assisted delivery methods) compared to the CEI protocol, but has little effect on hemodynamic conditions or drug dosage

Childbirth pain is one of the most painful experiences for women and affects physiological pain as well as maternal satisfaction. Given the importance and emphasis of the World Health Organization on physiological delivery, the importance of inducing painless delivery for greater comfort and satisfaction of mothers is highlighted. Therefore, it is necessary to provide a practical and possible analgesia along with improving maternal satisfaction and reducing side effects on the mother and fetus. Among the various methods of pain control during childbirth, the epidural method has the most acceptance and efficiency in terms of pain control and is recommended in all written guidelines (1).

Epidural analgesia is a very effective and a common technique in painless delivery that is recommended to be performed via programmed intermittent epidural boluses (PIEB) and continuous epidural infusion (CEI). In recent years, the superiority of PIEB over other techniques has been shown. In this way, a lower dose of blues medicine causes better analgesia and better satisfaction.

However, more studies are needed to confirm these findings as well as to find the ideal drug combination (2-5). Most studies have performed these two methods of analgesia in painless delivery (PIEB, CEI) with combinations of Bupivacaine, Ropivacain, Levobupivacaine and Fentanyl (2). One of the important features and benefits of Ropivacain is the separation of motor and sensory blocks. But it has less effect on reducing pain at higher doses. Ropivacain is capable of blocking the sodium channels and reducing the cardiac conduction, resulting in longer QRS and QT interval, but this reduction is less than that of bupivacaine (3).

Dexmedetomidine is an A2 agonist for A2 adrenergic receptors that has 8 times more affinity than clonidine, which provides adequate sedation and analgesia without the effects of respiratory depression even at high doses (4-6), and intensifies analgesic effects of a local anesthetic agent without increasing the incidence of side effects (7, 8). Dexmedetomidine causes a predictable dose-dependent change in patients' hemodynamics by lowering catecholamine levels after surgery, as well as reducing delirium and agitation. In the cardiovascular system, dexmedetomidine is capable of producing a biphasic response to blood pressure, resulting in a brief hypertension followed by hypotension. Dexmedetomidine reduces cerebral blood flow and reduces the metabolic need for oxygen. Side effects of dexmedetomidine include: hypotension, hypertension, bradycardia, dry mouth, atrial fibrillation, chills, pleural effusion, hyperglycemia, atelectasis, hypocalcemia and acidosis (9). In addition to intravenous use for epidural epidural and neuroaxial blocks, their fewer side effects have been confirmed in experimental and clinical studies (10, 11). Therefore, the aim of this study was to compare the effect of PIEB and CEI in painless delivery of Dexmedetomidine, and Ropivacain.

## Methods

This randomized clinical trial was performed on term female candidates for epidural anesthesia. Patients with inclusion and exclusion criteria were included in the study. Inclusion criteria include: term woman with a gestational age greater than or equal to 37 weeks, cervical dilatation> 4 cm, age between 18 and 45 years, weight between 55 to 85 kg, spontaneous labor or induced labor with request for epidural analgesia and ASA = I-II. Exclusion criteria include: Patients with a history of severe cardiopulmonary disease, bradycardia, contraindications to epidural anesthesia or cesarean section (multifetal pregnancy, gestational age under 37 weeks with preeclampsia), patient dissatisfaction, failure of epidural block, drug addiction and height < 150 cm. Sample size

The sample size was calculated based on a study by Capogna et al. (12). Considering the coefficient of 0.5 and the study power of 80%, the number of sample sizes for the design was calculated to be 48 individuals using the following formula. Taking into account 15% precipitation, the final sample size was 58 people (29 in each group).

**Procedure: **At the beginning of the project, written consent was obtained from patients. Patients were then subjected to PIEB or CEI using a random number table in one of the two groups. In each group, after performing standard monitoring (ECG, POM, NIBP), taking a venous line with 20 angiocatheter and administering 5cc / kg lactate ringer solution, the patient was placed in a sitting position and an 18-gauge epidural needle from L2-L3 or L3-L4 was entered in the epidural space up to 4 cm using the loss of resistance to saline 3 methods as Cephalad.

Five minutes after injection, a total of 3 cc of Dexmedetomidine) 0.5 µg/ml (and Ropivacaine 0.1% was injected. Furthermore, 5 ml was injected as a loading dose of Dexmedetomidine 0.5 µg /ml and Ropivacaine 0.1%. Then, in the PIEB method, the first bolus dose of 5 cc of the drug combination was injected every 60 minutes and 60 minutes after loading dose. In the CEI method, the amount of 5 cc per hour of infusion was administered immediately after the initial dose as Maintenance by B-braun infusion pump. Bolus doses were administered by a single individual using manual pumps. Also in both groups, Patient Control Epidural Analgesia (PCEA) was administered using a secondary infusion pump with the same drug combination and a dose of 3 ml bolus for sudden and severe pain, if the pain score was higher than 5. Maternal satisfaction, analgesia, Apgar score, instrumental delivery, duration of labor, maternal side effects (nausea, vomiting, pruritus, and hypotension), hemodynamic conditions and vital signs, delivery method and dosage of drug used in each two groups were recorded.

Data were collected and recorded by a person who was not aware of the study grouping. Maternal satisfaction and all information collected in each patient were recorded in a pre-designed questionnaire and form. Hypotension was considered as a drop in blood pressure of 20% of baseline blood pressure or blood pressure less than 90/60. Maternal satisfaction and analgesia were recorded by numeric analogue scale (NAS) in which 0 was considered completely dissatisfied and 10 were completely satisfied. Also, movement block was assessed by Bromage Score (0 = no motor loss, 1 = inability to flex hip, 2 = inability to flex hip and knee, 3 = inability to flex hip knee and ankle).

**Data analysis: **The results were expressed as mean and standard deviation (Mean ± SD) for quantitative variables and as a percentage for stratified qualitative variables. T-test or ANOVA was applied to compare quantitative variables and chi-square test was used to compare qualitative variables. Significance level was considered less than 0.05. SPSS software Version 23 was applied for statistical analysis of data.

**Ethical considerations: **The purpose of the study was described for all research units and written consent was obtained from them. The information of all patients was kept confidential by the project manager. In all stages of research, all ethics statements in Helsinki research and ethics research committees of the University of Medical Sciences were considered. The study was carried out after approval by the Research Council of the Medical School and receiving the code of ethics with the number IR.IUMS.FMD.REC.1399.397.

## Results

A total of 60 patients were included in this study, of which 30 patients underwent epidural anesthesia by PIEB method and 30 patients underwent CEI. The mean age of patients in the two groups was 24.1 ± 5.26 years and 25.5 ± 5.74 years, respectively, which did not show a significant difference between the two groups (P = 0.329).

In the two groups of PIEB and CEI, the mean BMI of patients was found to be 29.83 ±4.15 kg / m2 and 30.21±3.6 kg / m2, respectively, which did not show a significant difference between the two groups (P = 0.7). The frequency of gravid one was 83.3% and 73.3%, respectively, the frequency of gravid 2 was 10% and 16.7% and the frequency of gravid three was found to be 6.7% and 10%, respectively, which did not show any difference between the two groups (P = 0.64).

 Parity frequencies of zero were 90% and 96.7%, respectively. Parity frequency of one-fold was equal to 6.7% and 3.3% and parity frequencies of double were 3.3% and 0%, which did not show any difference between the two groups (P = 0.495).

The frequency of non-abortion was 90% and 86.7%, respectively. The frequency of one-time abortion was 6.7% and 13.3% and the frequency of two-time abortion was 3.3% and 0%, which did not reveal a significant difference between the two groups (P = 0.431). In the two groups of PIEB and CEI, the mean gestational age of the patients in the two groups was found to be 38.97 ± 0.99 and 39.1±0.84 weeks, respectively, which did not demonstrate a significant difference between the two groups (P = 0.579).

In the two groups of PIEB and CEI, the frequency of ASA-I was 53.3% and 60%, respectively, and the frequency of ASA-II was 46.7% and 40%, respectively, which did not show a difference between the two groups (P = 0.602). In PIEB and CEI groups, the prevalence of gestational diabetes was 13.3% and 10%, respectively. The frequency of hypertension was 6.6% and 0%, the frequency of hypothyroidism in both groups was 26.6% and the frequency of hyperthyroidism was 3.3% and 3.5%, respectively, which did not show a difference between the two groups (P = 0.9). In PIEB and CEI groups, the mean dilatation of patients in the two groups was 4.05 ±0.91 cm and 4.86±0.86 cm, respectively, which did not show a significant difference between the two groups (P = 0.427). The mean of effacement in PIEB and CEI groups was 48.67 ±9.99 and 49.67 ±10.66, respectively, which did not show a significant difference between the two groups (P = 0.709). Station status in all patients in the two groups was assessed to be equal to -3. In terms of side effects, no side effects such as nausea, vomiting, pruritus, hypotension or bradycardia were reported.

**Figure 1:**
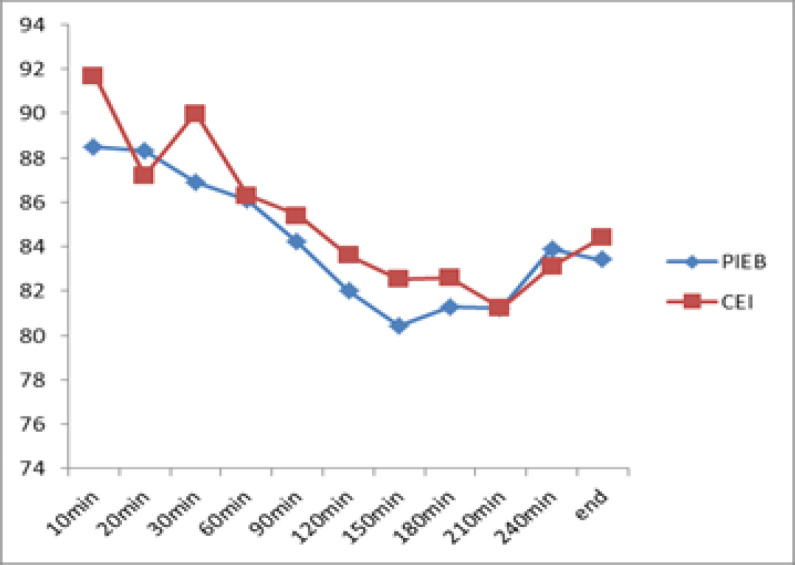
Changes in heart rate

Regarding the hemodynamic conditions, the trend of changes in systolic and diastolic blood pressure at minutes 0, 10, 20, 30, 60, 60, 90, 120, 150, 180, 210, 240 and at the end of labor in the two groups of PIEB and CEI were studied, indicating no significant difference in the trend of systolic and diastolic blood pressure changes (P = 0.456, P = 0.776; table 1). The study of changes in heart rate at different minutes in the two groups of PIEB and CEI did not show a significant difference.(p=0.224)(figure1)

**Figure 2:**
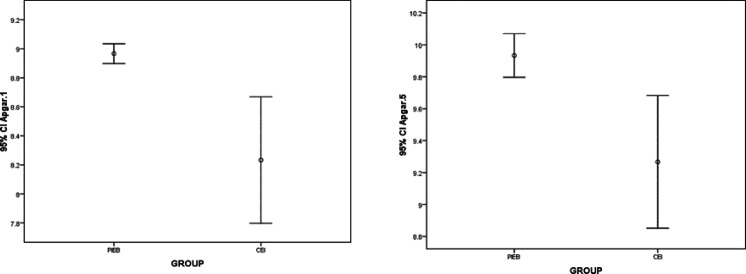
Average Apgar score of the baby

Apgar score in the first minute in PIEB and CEI groups was 8.97± 0.18 and 8.23±1.16, respectively, which was significantly higher in the first group (P = 0.001). Furthermore, the mean neonatal Apgar score in the fifth minute in the two groups was 9.93±0.36 and 9.27± 1.11, respectively, which was found to be significantly higher in the first group (P = 0.003; figure 2).

**Figure 3:**
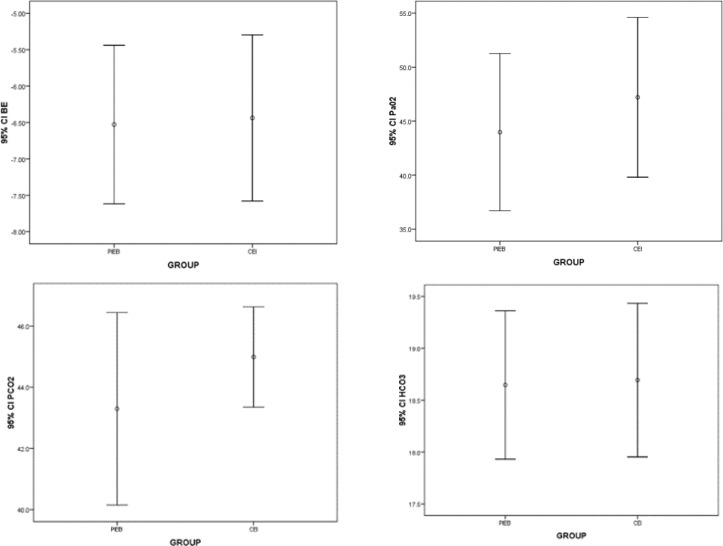
Mean BE, PaO2, HCO3 and PCO2

The mean Base Excess of the two groups) PIEB and CEI (were found to be -6.52± 2.91 and -643 ±3.05 (P= 0.903), respectively, followed by the mean arterial oxygen pressure (43.98 ±19.5and 47.21± 19.81 mm Hg; P = 0.527), mean HCO3 concentrations (18.64 ± 1.91 and 18.69 ± 1.97 mEq; P = 0.926) and mean carbon dioxide pressures (43.29 ± 8.41 vs 44.98 ± 4.38 mm Hg; P = 0.33). There was no significant difference between the two groups in terms of BE, PaO2, HCO3 and PCO2 (P> 0.05), (figure 3).

**Table 1:** Changes in systolic and diastolic blood pressure in the two groups

**Characterization**		**PIEB**	**CEI**	**P-value**
**0 min**	**systolic**	125.27±11.08	127.43±11.38	0.458
	**diastolic**	76.6±4.47	75.97±4.71	0.595
**10 min**	**systolic**	123.53±9.61	125.3±9.26	0.472
	**diastolic**	73.93±6.31	73.93±6.31	1.0
**20 min**	**systolic**	120.53±10.58	120.53±10.58	1.0
	**diastolic**	72.27±7.47	72.1±7.97	0.934
**30 min**	**systolic**	123.53±15.9	122.57±15.28	0.811
	**diastolic**	73.3±7.17	74.09±6.55	0.371
**60 min**	**systolic**	123.03±10.81	122.83±9.26	0.939
	**diastolic**	75.03±7.76	75.6±5.84	0.75
**90 min**	**systolic**	120.7±9.46	123.8±9.28	0.205
	**diastolic**	73.7±9.22	75.9±10.23	0.386
**120 min**	**systolic**	120.07±9.68	121.53±9.23	0.55
	**diastolic**	74.77±8.58	75.83±8.34	0.628
**150 min**	**systolic**	120.07±11.13	120.43±11.11	0.905
	**diastolic**	70.86±7.36	71.96±4.82	0.521
**180 min**	**systolic**	119.81±8.35	112.07±31.5	0.221
	**diastolic**	74.33±7.99	69.47±7.34	0.25
**210 min**	**systolic**	122.2±5.36	121.32±5.9	0.617
	**diastolic**	74.68±3.99	73.45±4.18	0.325
**240 min**	**systolic**	123.4±5.44	116.14±6.73	0.204
	**diastolic**	75.29±4.76	76.6±4.27	0.359
**The end of delivery**	**systolic**	121.23±8.53	122.07±8.78	0.711
**diastolic**	73.03±6.48	73.07±6.02	0.984

The mean blood pH in the two groups of PIEB and CEI groups was 7.23 ± 0.04 and 7.22 ±0.04, respectively, which was not different between the two groups. (P = 0.5). In the two groups of epidural anesthesia, 6.7% of delivery was reported to be linked to cesarean section and 93.3% was also related to normal vaginal deliveries, indicating no difference in delivery method between the two groups (P = 1.0). The frequency of use of instrumental delivery (vacuum) was 6.7% and 26.7%, respectively, which was found to be significantly lower in the group of PIEB (P = 0.038).

**Figure 4:**
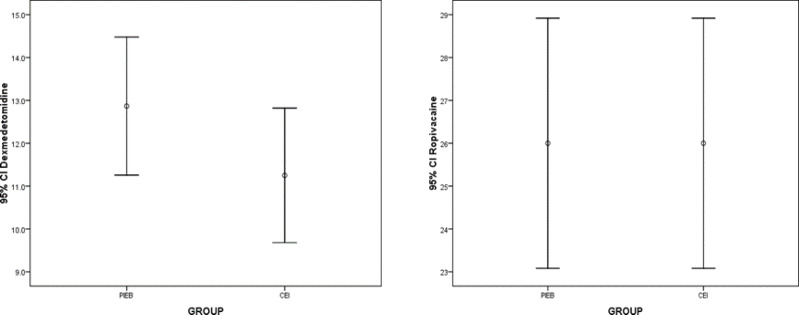
Mean consumption of dexmedetomidine and ropivacaine

The mean amount of dexmedetomidine in the two groups was 12.86±4.31 and 11.25 ±4.2, respectively, which was not different between the two groups (P = 0.147). The mean amount of ropivacaine in the two groups was 25.13 ± 7.36 and 26.42 ± 5.98, respectively, which was not different between the two groups (P = 0.426) (figure 4).

The frequency of secondary analgesia in PIEB and CEI groups was 3.3% and 0%, respectively, which was not different between the two groups (P = 0.998). Based on the evaluation of motor block based on Bromage Score, no motor block cases were reported in either group. The mean level of satisfaction based on NAS score in the two groups was 1.98 ± 5.2 and 0.9 ± 2.87, respectively, which was significantly higher in the group under PIEB (P = 0.002). The mean duration of the first phase of labor in the two groups was 4.27 ±0.27 and 4.48 ±0.57 hours, respectively, which was significantly shorter in the group under PIEB (P = 0.015). The mean duration of the second phase of labor in the two groups was 0.78±0.43 ± and 0.72±0.43 hours, respectively, which was not different between the two groups (P = 0.6).

## Discussion

Continuous epidural infusion or CEI has been an optimal and acceptable technique for inducing epidural anesthesia for many years. However, some other programs and protocols such as scheduled intermittent epidural injections or PIEB, especially in the management of labor pains, have opened their special place in recent years. However, it remains unclear which of these methods is associated with better outcomes for mother and infant and ultimately higher maternal satisfaction (13). Therefore, the aim of the present study was to compare the two programs of PIEB and CEI for painless delivery and to evaluate its outcome of using dexmedetomidine and ropivacaine. What were considered as the outcomes of the study included maternal hemodynamic changes, neonatal infancy at birth, possible postpartum side effects, duration of labor phases, delivery method and use of assisted delivery methods, and finally maternal satisfaction with each method.

What was obtained in the present study and in line with previous studies was that, firstly, the Apgar score of the infant in the PIEB method showed more improvement, secondly, the use of assisted delivery tools such as vacuum in the PIEB method was reduced and also the duration of the first phase of labor. This method also showed a greater decrease than CEI. However, the hemodynamic condition of the patients in the two groups, including vital signs and arterial analysis, was maintained in a stable state. As a result, patients in the group undergoing PIEB were associated with a higher level of satisfaction with the delivery process and induction of epidural anesthesia than patients undergoing CEI protocol. In other words, improving the vitality of newborns at birth and facilitating the delivery process (improving the length of the delivery phase and not using assisted delivery methods) had led to higher patient satisfaction with such a protocol. Despite the differences in different aesthetic compounds for anesthesia, the PIEB method still showed superiority.

A systematic review and meta-analysis by Huang et al. reviewed 20 clinical trial studies comparing the outcomes of the PIEB and CEI programs and patient satisfaction with the use of rupivacaine for maintenance of anesthesia. Their results showed that the use of PIEB with ropivacaine was significantly associated with increased patient satisfaction, decreased anesthetic substance and decreased second stage of labor compared to CEI. This technique has also been associated with a reduction in the incidence of motor block compared to CEI. There was no difference between the two methods in terms of auxiliary bolus delivery method (14). However, for some findings, some differences were observed between this comprehensive assessment and the present study. But the PIEB method was superior to the CEI method in terms of birth defects. In the present study, cases of motor block were not reported at all, which indicates the high efficiency and safety of this protocol.

A cohort study by Holgado et al. compared CEI with 0.2% ropivacaine + 100-μg fentanyl initial bolus versus PIEB+PCEA with 0.1% ropivacaine + 2 μg mL^-1^ fentanyl in primiparous women. The primary outcome was mode of delivery. In each group, 221 people were entered in the evaluation. The results of logistic regression model showed that PIEB + PCEA group was associated with fewer cases of cesarean section and reduced cases of instrumental deliveries. The second phase of labor was no different between the two groups. Also, the total dose of ropivacaine was much lower in the PIEB + PCEA group. There was no relationship between the occurrence of mild motor block and increased use of PCEA in the PIEB

+ PCEA group. This method was also associated with increased satisfaction of pregnant women, which was very similar to our study (15). Haidl et al., 150 women in the labor course were randomly evaluated by PIEB + PCEA vs CEI + PCEA using an epidural solution containing adrenaline. In a study by Haidl et al. in 2020, 150 women in the labor course were randomly assigned to PIEB + PCEA or CEI + PCEA. The drug regimen in both groups included bupivacaine and fentanyl. There was no difference between the two groups in terms of hourly dose of the drug. There was significant difference between the two groups in terms of the number of successfully administered PCEA boluses. Also, no significant difference was found between the two groups in terms of pain score, motor block, maternal satisfaction and the need for anesthesiologist intervention, which was in line with the present study (16). In a study by Song et al., pregnant women with a pain score above 5 and cervical dilatation below 5 cm were randomly assigned to the EP + CEI, DPE + CEI, or DPE + PIEB protocols for labor anesthesia. First, adequate labor anesthesia in the DPE + CEI and DPE + PIEB methods was much faster than the EP + CEI method. The use of DPE technique with PIEB was associated with the lowest number of PCEA boluses and the lowest number of ropivacaine use, but no difference between the methods in terms of pain score, delivery method, Apgar score, side effects and patient satisfaction, which was in stark contrast to those of the present study (17).

In the study by Fidkowski et al., evaluated continuous epidural infusion (CEI) to 2 PIEB regimens for labor analgesia. First, no significant difference was found in pain scores between the two groups. Dermatomal sensory level, degree of motor block and patient satisfaction were not different between the two groups (18). In a 2019 study by Riazanova et al., 84 primiparous women underwent epidural anesthesia using either the PIEB or CEI protocol. The anesthesia method was not related to the dynamic parameters of labor progression and fetal status. However, the amount of anesthetic used in the PIEB group was less than the CEI protocol. The first bolus in the PIEB group was found to be significantly longer than the CEI group, which was not consistent with the present study (19).

Another study demonstrated that the rate of assisted delivery, pain incidence, and successful rate of PCEA were much lower in the PIEB group and patient satisfaction was found to be much higher in the PIEB group than in the CEI group. No significant difference was found between the two methods in terms of side effects, which was quite similar to the results of the present study (1). It seems that significant differences in the results of different studies can be influenced by various parameters such as the characteristics of inclusion criteria, sample size of the subjects and different anesthesia protocols used in the trial.

The PIEB method is associated with further improvement in neonatal (Apgar score) and maternal outcomes (reduction in the duration of the first phase of labor and no need to use assisted delivery methods) compared to the CEI protocol, but has little effect on hemodynamic conditions or drug dosage. Further improvement of maternal and neonatal conditions following the use of PIEB method will eventually lead to greater maternal satisfaction.

## References

[1] Xu J, Zhou J, Xiao H (2019). a systematic review and meta-analysis comparing programmed intermittent bolus and continuous infusion as the background infusion for parturient-controlled epidural analgesia. Sci Rep.

[2] Gogoi S, Saikia D, Dey S (2022). Addition of clonidine or dexmedetomidine with bupivacaine to prolong caudal analgesia in children undergoing infraumbilical surgery. Cureus.

[3] Mostafa MF, Hamed E, Amin AH, Herdan R (2021). Dexmedetomidine versus clonidine adjuvants to levobupivacaine for ultrasound‐guided transversus abdominis plane block in paediatric laparoscopic orchiopexy: Randomized, double‐blind study. Eur J Pain.

[4] Nie Y, Liu Y, Luo Q, Huang S (2014). Effect of dexmedetomidine combined with sufentanil for post- caesarean section intravenous analgesia: a randomised, placebo-controlled study. Eur J Anaesthesiol.

[5] Naveen P, Gupta VK, Agarwal A (2021). Comparative Study for Anaesthetic Quality with the Addition of Clonidine, Fentanyl or Dexmedetomidine to 0 5℅ Ropivacaine in Supraclavicular Brachial Plexus Block at a Tertiary Hospital. J Clin Anesthes Res.

[6] Yousef AA, Salem HA, Moustafa MZ (2015). Effect of mini-dose epidural dexmedetomidine in elective cesarean section using combined spinal-epidural anesthesia: a randomized double-blinded controlled study. J Anesth.

[7] Wangping Z, Ming R (2017). Optimal dose of epidural dexmedetomidine added to ropivacaine for epidural labor analgesia: a pilot study. Evid Based Complement Alternat Med.

[8] Zhao Y, Xin Y, Liu Y, Yi X, Liu Y (2017). Effect of epidural dexmedetomidine combined with ropivacaine in labor analgesia: a randomized double-blinded controlled study. Clin J Pain.

[9] Kaur M, Singh PM (2011). Current role of dexmedetomidine in clinical anesthesia and intensive care. Anesth Essays Res.

[10] Vieira AM, Schnaider TB, Brandão AC (2004). Epidural clonidine or dexmedetomidine for post–cholecystectomy analgesia and sedation. Rev Bras Anestesiol.

[11] Rahimzadeh P, Imani F, Faiz SHR (2016). Adding Intra-Articular Growth Hormone to Platelet Rich Plasma under Ultrasound Guidance in Knee Osteoarthritis: A Comparative Double-Blind Clinical Trial. Anesth Pain Med.

[12] Salim R, Nachum Z, Moscovici R, Lavee M, Shalev E (2005). Continuous compared with intermittent epidural infusion on progress of labor and patient satisfaction. Obstet Gynecol.

[13] Loubert C, Hinova A, Fernando R (2011). Update on modern neuraxial analgesia in labour: a review of the literature of the last 5 years. Anaesthesia.

[14] Huang R, Zhu J, Zhao Z, Wang B (2021). The effect of programmed intermittent epidural bolus compared with continuous epidural infusion in labor analgesia with ropivacaine: a meta- analysis of randomized controlled trials. Ann Palliat Med.

[15] Holgado CM, Girones A, Tapia N, De Molina-Fernandez MI, Anez C (2020). Labor outcomes with epidural analgesia: an observational before-and-after cohort study comparing continuous infusion versus programmed intermittent bolus plus patient-controlled analgesia. Minerva Anestesiol.

[16] Haidl F, Arne Rosseland L, Rørvik AM, Dahl V (2020). Programmed intermittent boluses vs continuous epidural infusion in labor using an adrenaline containing solution: A randomized trial. Acta Anaesthesiol Scand.

[17] Song Y, Du W, Zhou S (2021). Effect of dural puncture epidural technique combined with programmed intermittent epidural bolus on labor analgesia onset and maintenance: a randomized controlled trial. Anesth Analg.

[18] Fidkowski CW, Shah S, Alsaden MR (2019). Programmed intermittent epidural bolus as compared to continuous epidural infusion for the maintenance of labor analgesia: a prospective randomized single-blinded controlled trial. Korean J Anesthesiol.

[19] Riazanova OV, Alexandrovich YS, Guseva YV, Ioscovich AM (2019). A randomized comparison of low dose ropivacaine programmed intermittent epidural bolus with continuous epidural infusion for labour analgesia. Rom J Anaesth Intensive Care.

